# Loss of outer arm dynein components produce specific and separable phenotypes in zebrafish

**DOI:** 10.1186/2046-2530-1-S1-O17

**Published:** 2012-11-16

**Authors:** R Burdine, CE Slagle

**Affiliations:** 1 Princeton University, USA

## 

Structural and functional defects in cilia lie at the heart of several human genetic disorders, including polycystic kidney disease, male sterility, retinal degeneration, respiratory complications, and organ laterality defects. It is therefore crucial to study various aspects of cilia biology so as to better understand the exact molecular causes of these disorders. In our current study, we utilize a novel zebrafish mutant called *flanders* to study the relationship between organ laterality defects and kidney cyst development. *flanders* is expressed in both ciliated Kupffer’s vesicle, important for left-right patterning, and in the pronephros/kidney. However, unlike other laterality mutants we have studied, *flanders* homozygotes display a strong laterality phenotype without an associated strong kidney cyst phenotype, despite the *flanders* gene showing high homology to a structural component the outer arm dynein complex. To determine why *flanders* does not appear to be critical in pronephric cyst formation, we looked for other genes that may function redundantly in this tissue. We show that a *flanders* paralog, *ccdc114*, plays a minor role in organ laterality but interacts with *flanders* during cyst development. Loss of flanders or ccdc114 reduces cilia beat frequencies in both Kupffer’s vesicle and the kidney even though each protein only produces phenotypes thought to originate in one of these tissues. Our data suggest that outer arm dynein complex composition may regulate a functional difference between motile cilia in Kupffer’s vesicle and those in the kidney. We will discuss our ongoing work on this mechanism.

